# Reduced toxicity conditioning and a high CD34^+^ cell dose can achieve full donor chimerism in DOCK8 deficiency

**DOI:** 10.1016/j.jacig.2023.100106

**Published:** 2023-03-28

**Authors:** Ambreen Pandrowala, Ajay Narayan Sharma, Manasa Kakunje, Minnie Bodhanwala, Prashant Hiwarkar

**Affiliations:** a Department of Blood and Marrow Transplantation, Mumbai, India; b Department of Pediatrics, Bai Jerbai Wadia Hospital for Children, Mumbai, India

**Keywords:** DOCK8 deficiency, bone marrow transplantation, HSCT, chimerism

## Abstract

**Background:**

Biallelic mutations in the dedicator of cytokinesis 8 (*DOCK8*) gene were identified as the cause of combined immunodeficiency in 2009. Survival rates without hematopoietic stem cell transplant in patients with DOCK8 deficiency decline from 87% at 10 years to 33% at 30 years. Hematopoietic stem cell transplant is therefore the recommended treatment for cure of DOCK8 deficiency. However, patients with DOCK8 deficiency have multiple infectious comorbidities; hence, they cannot tolerate myeloablative conditioning. Reduced intensity conditioning reduces the risk of transplant-related mortality but increases the possibility of mixed chimerism. Mixed chimerism in children with immunodeficiency increases the risk of autoimmunity and the need for long-term immunoglobulin infusion.

**Objective:**

Here we have sought to devise a strategy for reducing the possibility of mixed chimerism without increasing the risk of transplant-related mortality.

**Methods:**

To balance the risk of transplant-related mortality and mixed chimerism, we used treosulfan-based reduced toxicity conditioning with a high CD34^+^ cell dose and differential T-cell capping for HLA-matched and haploidentical transplants.

**Results:**

We are able to report that by using the aforementioned novel strategy, we achieved excellent transplant outcomes in the first cohort of high-risk patients with DOCK8 deficiency from India.

**Conclusion:**

High CD34^+^ cell dose and reduced toxicity conditioning can achieve full donor chimerism in DOCK8 deficiency.

## Introduction

DOCK8 deficiency manifests as combined immunodeficiency with recurrent viral, bacterial, and fungal infections, as well as features of immune dysregulation such as eczema, allergies, lymphoproliferation, and autoimmunity. In immune dysregulatory disorders such as Wiskott-Aldrich syndrome, mixed chimerism increases the risk of posttransplant autoimmunity.^1^ There is a lack of definite evidence on whether mixed chimerism in DOCK8-deficient patients increases the risk of posttransplant autoimmunity. However, DOCK8-deficient patients with mixed chimerism continue to require long-term immunoglobulin infusion.^2^ Therefore, a recent study of lineage-specific chimerism in patients with DOCK8 deficiency advocated aiming for complete donor chimerism with reduced intensity conditioning.^2^ Reduced intensity conditioning increases the likelihood of mixed chimerism, whereas myeloablative conditioning in children with immunodeficiencies increases the risk of transplant-related mortality.^3^ To strike a balance between conditioning toxicity and chimerism, we adopted a strategy of reduced toxicity conditioning and high CD34^+^ cell dose. Here, we report the transplant outcomes and immune reconstitution achieved in the first Indian cohort of patients with DOCK8 deficiency by using the novel strategy.

## Results and discussion

A total of 4 consecutive children with DOCK8 deficiency underwent hematopoietic stem cell transplants (HSCTs) from 2019 to 2021 according to the institutional standard of care transplant protocol. Their age at transplant ranged from 1.5 years to 12 years (median age 3.1 years). The children's pretransplant clinical features are summarized in Table I. Before the transplant, all infections were treated aggressively. Failure to thrive was present in all 4 children. Patients 3 and 4 had no respiratory tract symptoms; however, a chest computed tomography scan was suggestive of infective foci, and bronchoalveolar lavage isolated *Streptococcus pneumoniae* in patient 4 and *Pseudomonas aeruginosa* and *S pneumoniae* with positive galactomannan in patient 3. Patient 2 developed ataxia with signs of raised intracranial tension before starting conditioning. His brain magnetic resonance imaging scan (see Fig E1 in the Online Repository at www.jaci-global.org) showed cerebellar abscesses, which were drained. Pus from the abscess grew *Nocardia* species. The patient was treated with meropenem and cotrimoxazole for 6 weeks before starting conditioning, as well as with amoxicillin-clavulanate and ciprofloxacin during the transplant. All of the patients had eczema, and their eczema was managed with once-weekly methotrexate in 2 severe cases for better control before the transplant. DOCK8 deficiency was diagnosed with genetic mutation and flow cytometry.

**Table I:** Demography, pretransplant infections, pretransplant allergies, transplant characteristics, and posttransplant follow-up of patients with DOCK8 deficiency

Patient characteristic	Patient			
	1	2	3	4
Age at diagnosis	2 y	11 y	6 mo	1 y
Mutation	Homozygous 3' splice site variation in intron 16	Homozygous deletion exons 1-47	Homozygous 3' splice site variation in intron 16	Homozygous deletion exons 1-13
Pretransplant infections				
Sinopulmonary infections	H1N1, RSV	Bilateral otitis media, *Pseudomonas aeruginosa,* and *Klebsiella pneumoniae*	*P aeruginosa*, *S pneumoniae*, possible *Aspergillus*	RSV A and B, *S pneumoniae*
Skin infections	Disseminated molluscum contagiosum	*Staphylococcus aureus*	HSV, disseminated molluscum, fungal	
Gut infections			*Cryptosporidium* (no evidence of liver involvement)	
CNS infections		*Nocardia* cerebellar abscesses	Epileptiform discharges in temporal lobe ?HSV	
Severity of eczema (SCORAD score)	28.9	61	81.9	50.25
Pretransplant allergies and/or eczema				
Eczema	Yes	Yes Taking oral methotrexate	Yes	Yes Taking oral ciclosporin followed by methotrexate
Allergies	None	None	None	Food
Transplant characteristic				
Patient age at transplant	2.6 y	12.3 y	2.9 y	3.3 y
Donor	Matched related donor	Matched related donor	Haploidentical father	Partially matched (A and C antigen mismatch) unrelated donor
Stem cell source	PBSC	PBSC	PBSC	PBSC
Stem cell processing	None	None	TCR αβ and CD45RA depletion	TCR αβ and CD45RA depletion
Conditioning	Fludarabine, 160 mg/m^2^ Treosulfan, 42 g/m^2^ Thiotepa, 10 mg/kg Alemtuzumab, 0.5mg/kg	Fludarabine, 160 mg/m^2^ Treosulfan, 42 g/m^2^ Alemtuzumab, 0.9 mg/kg	Fludarabine, 160 mg/m^2^ Treosulfan, 42 g/m^2^ Thiotepa, 10 mg/kg rATG, 7.5 mg/kg	Fludarabine, 160 mg/m^2^ Treosulfan, 42 g/m^2^ Thiotepa, 10 mg/kg rATG, 7.5 mg/kg
CD34 cell dose	10.6 × 10^6^/kg	14 × 10^6^/kg	30 × 10^6^/kg	19 × 10^6^/kg
T-cell dose	5 × 10^8^/kg	5 × 10^8^/kg	αβ-0	αβ-4.3 × 10^4^/kg
GvHD prophylaxis	CsA and MMF	CsA and MMF	CsA	CsA
Issues during transplant	Upper airway RSV infection	?Nocardia reactivation with respiratory involvement	Engraftment syndrome, adenoviremia	Engraftment syndrome
Neutrophil engraftment	Day +11	Day +13	Day +10	Day +19
Platelet engraftment	Day +9	Day +10	Day +6	Day +10
Acute GvHD	Nil	Nil	Nil	Nil
Discharge	Day +25	Day +34	Day +31	Day +38
Posttransplant follow-up				
Infection	MRSA skin infection	1 episode of lower respiratory tract infection	None	None
Immunosuppression	Day +130	Day +175	Day +84	Ongoing (MMF and low-dose prednisolone for posttransplant autoimmune hemolytic anemia)
Chimerism on last follow-up	100% Day +365	100% Day +365	100% Day +365	95% Day +362
Follow-up status	Day +912 Well, with resolution of eczema and infections	Day+833 Well, with resolution of eczema and infections	Day +454 Well, with resolution of eczema and infections	Day +362 Well, with resolution of eczema, infections and allergies

*CNS,* Central nervous system; *CsA*, ciclosporin A; *HSV*, herpes simplex virus; *MMF*, mycophenolate mofetil; *MRSA*, methicillin-resistant *Staphylococcus aureus*; *PBSC*, peripheral blood stem cell; *rATG*, rabbit antithymocyte globulin; *RSV*, respiratory syncytial virus; *SCORAD*, SCORing for Atopic Dermatitis; *TCR*, T-cell receptor.

All of the children received fludarabine and treosulfan–based conditioning with or without thiotepa. Patient 2 did not receive thiotepa because of severe lymphopenia. Serotherapy was either alemtuzumab for matched donors or Genzyme ATG for haploidentical donors. Patient 4 was an adopted child with no matched donors; hence, an unrelated haploid donor was used with T-cell receptor αβ and CD45RA depletion. Patients 3 and 4 both received CD45RO cells on day 1 after the transplant (day +1). The median CD34^+^ cell dose was 16.5 million cells/kg (range 10.6-30 million cells/kg) with the T-cell dose in the HLA-matched transplant capped at 5 × 10^8^ cells/kg and the αβ T-cell dose in the haploidentical transplant capped at 1 × 10^5^ cells/kg. For graft-versus-host disease (GvHD) prophylaxis, we used ciclosporin and mycophenolate mofetil in matched donors and only ciclosporin in haploidentical donors.

All of the children had rapid engraftment, median neutrophil engraftment at day +12, and platelet engraftment at day +10. None of them developed acute GvHD.

**Fig 1:**
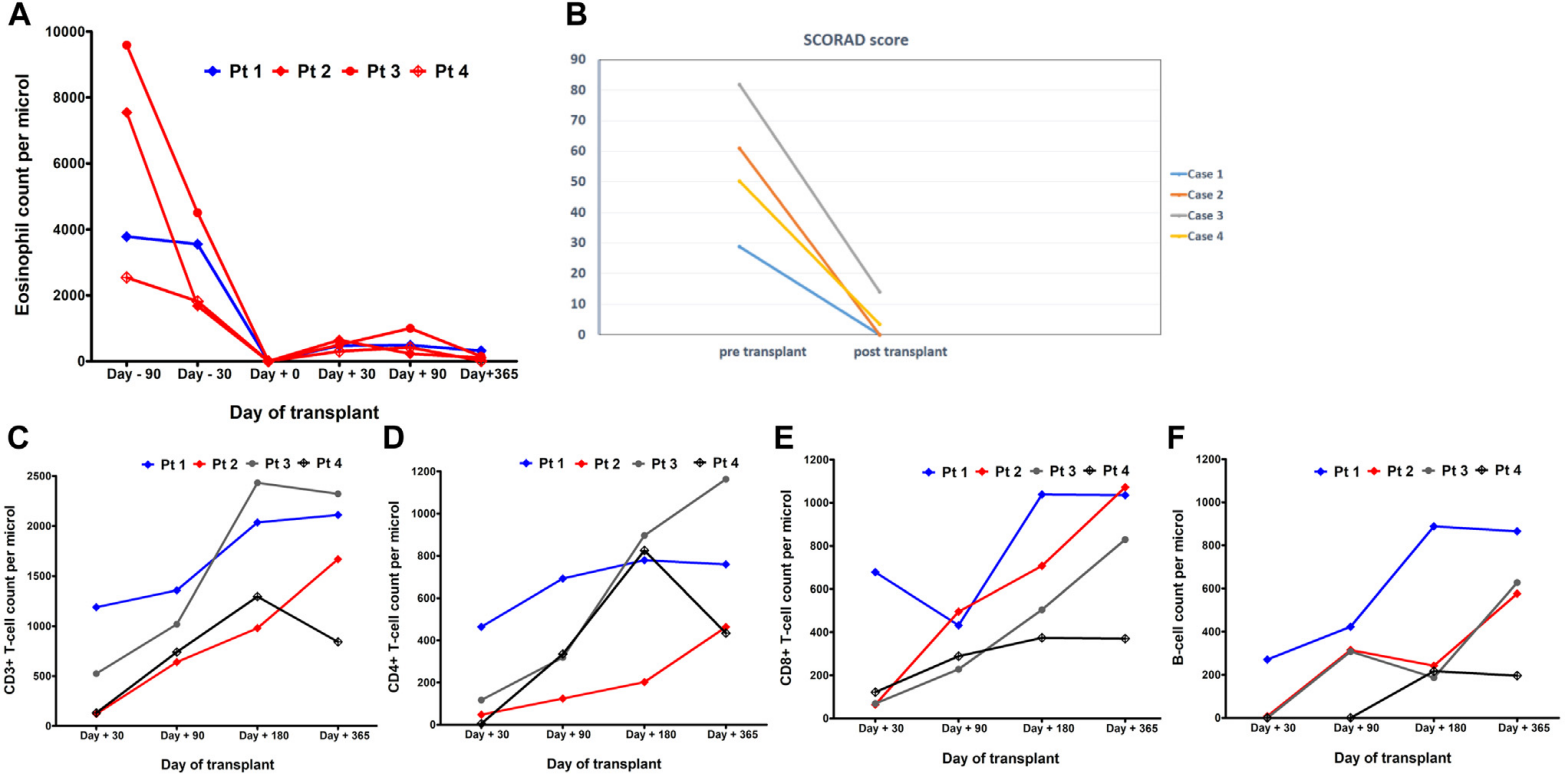
**A** and **B,** Eosinophil count and SCORing for Atopic Dermatitis (SCORAD) before and 2 months after transplant. **C-F,** Rapid expansion of immune cells (CD3^+^ T cells [**C**], CD4^+^ T cells [**D**], CD8^+^ T cells [**E**], and B cells [**F**]) following transplant.

After transplant, all of the children had significant resolution of their eczema. Absolute eosinophil count was significantly elevated in all 4 patients before the transplant (Fig 1, *A*). Patient 4 had lichenification of the skin before the transplant. The lichenified skin became normal on follow-up after the transplant (see Fig E2 in the Online Repository at www.jaci-global.org). Eczema resolved in all cases, with a resolution of molluscum contagiosum and skin fungal infection and normalization of eosinophil count (Fig 1, *A*). SCORing for Atopic Dermatitis (SCORAD) before and 2 months after transplant is shown in Fig 1, *B*. Except for 1 child who needed a repeat admission for a lower respiratory tract infection, all of the children had an uneventful posttransplant course with weight gain and resolution of eczema.

Immunologic recovery is shown in Fig 1. Lymphocyte subsets, including CD3^+^ T cells, CD4^+^ T cells, CD8^+^ T cells, B cells, and natural killer cells were measured at 1, 3, 6, and 12 months after transplant. Rapid immune reconstitution of T cells was seen following transplantation, with a median CD3^+^ cell count of 2195 cells/mm^3^ at 3 months after transplant (range 1060-2590 cells/mm^3^) (Fig 1, *C*-*E*). All of the children except 1 received rituximab before transplant to reduce the risk of Epstein-Barr virus–related lymphoproliferative disorder. The use of rituximab resulted in a slower B-cell recovery (Fig 1, *F*). The molluscum skin lesions had resolved on follow-up, with no new viral infections.

DOCK8 deficiency has poor overall survival without transplant and excellent outcomes after transplant. In 2011 Gatz et al reported the first 2 cases of HSCT in DOCK8-deficient children with complete resolution of disfiguring molluscum contagiosum in both patients.^4^ This paved the way for understanding that the unusual susceptibility in this cohort to viral skin infections was exclusively due to the immunodeficiency and not due to abnormality of non-hematopoietic cells related to DOCK8.^4^ Aydin et al published the largest cohort of patients with DOCK8 deficiency in 2015, establishing the natural history of the disease in the absence of HSCT.^5^ The median survival time was only 20 years, even with extensive supportive therapies. Other than infections, morbidities such as cerebral events, malignancy, vasculitis, and autoimmunity were also seen.^5^ Results of HSCT in a large international cohort of DOCK8-deficient patients were reported in 2019.^6^ A total of 81 patients from 22 centers were evaluated. Their median age was 9.7 years. They had an 84% survival, with reduced intensity conditioning resulting in superior survival compared with myeloablative conditioning (97% vs 78%), and 89% had more than 90% donor T-cell chimerism at the last follow-up.^6^ Another report showed improved quality of life and survival following HSCT versus in those who did not receive HSCT.^7^ Raedler et al published a cohort of 9 patients, 4 of whom had mixed chimerism.^2^ Of note, they used a reduced intensity conditioning in all but 1 patient, with a median CD34^+^ cell dose of 5.6 million cells/kg (range 2.4-14.1 million cells/kg). Peripheral blood stem cells were used as a stem cell source in 2 patients, and bone marrow was used in the remaining 7 patients. The immunologic outcome in patients with mixed chimerism showed mixed B-cell chimerism, possibly resulting in hypogammaglobulinemia and therefore long-term immunoglobulin infusion. Long-term immunoglobulin infusion further adds to the financial toxicity of treating patients with chronic diseases such as DOCK8 deficiency.

Therefore, to reduce the possibility of mixed chimerism, we used T-cell capping, thus allowing the infusion of high numbers of CD34^+^ cells. All patients received reduced toxicity conditioning with a high CD34^+^ cell dose, and all of the patients achieved at least 95% donor chimerism in the peripheral blood with normal immunoglobulin levels at the last follow-up. The resolution of eczema and infectious complications was quick, with good immune reconstitution on follow-up. All of the children in our cohort are doing well on follow-up, with no chronic GvHD. Our report suggests that excellent disease-free survival with full donor chimerism could be achieved in patients with DOCK8 deficiency by using reduced toxicity conditioning and novel methods of transplantation that allow grafting with high CD34^+^ cell dose.

**Key messages**

• Reduced toxicity conditioning allows the grafting of high-risk patients with DOCK8 deficiency with excellent outcomes.
• High CD34^+^ cell dose and reduced toxicity conditioning can achieve full donor chimerism in DOCK8 deficiency.
