# Supplementary material for: Reduced toxicity conditioning and a high CD34+ cell dose can achieve full donor chimerism in DOCK8 deficiency
Source: J Allergy Clin Immunol Glob. 2023 Mar 28;2(3):100106. doi: 10.1016/j.jacig.2023.100106 (PMC10510004; doi:10.1016/j.jacig.2023.100106)
Supplement: Supplementary Figures [file mmc1.pdf]

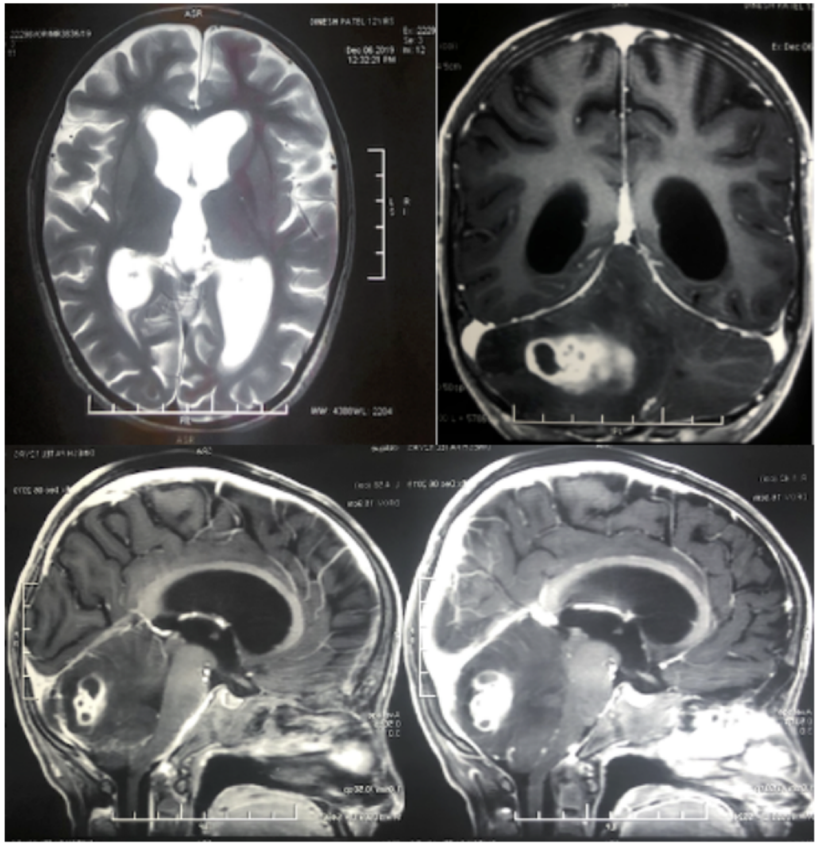

Supplemental figure 1 MRI images showing cerebellar abscesses in patient 2

(a) (b)

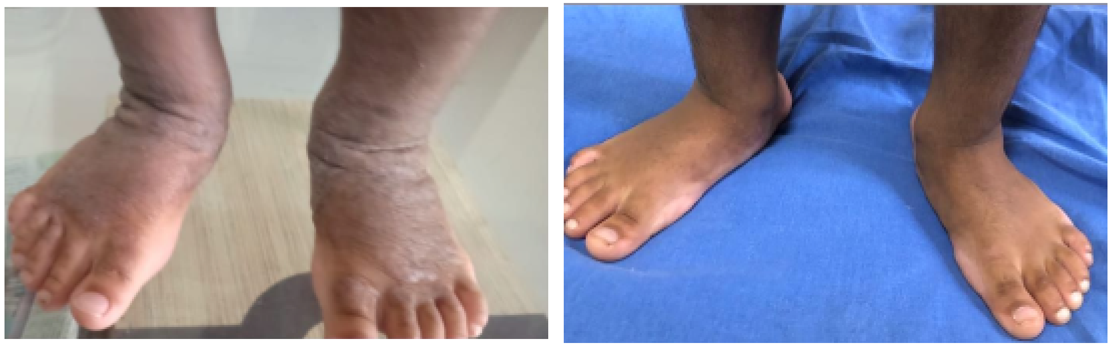

Supplemental figure 2 (a) showing lichenification of skin due to DOCK8 deficiency (b) Normal skin following transplant.
